# Causes and consequences of contrasting genetic structure in sympatrically growing and closely related species

**DOI:** 10.1093/aobpla/plv106

**Published:** 2015-09-02

**Authors:** Ivan Radosavljević, Zlatko Satovic, Zlatko Liber

**Affiliations:** 1 Faculty of Science, Department of Botany, Division of Biology, University of Zagreb, Marulicev trg 9a, HR 10000 Zagreb, Croatia; 2 Faculty of Agriculture, Department of Seed Science and Technology, University of Zagreb, Svetošimunska cesta 25, HR 10000 Zagreb, Croatia

**Keywords:** Hybridization, Mediterranean, population bottleneck, population genetics, *Salvia*, SSR

## Abstract

Gene flow, natural selection and genetic drift are processes that play a major role in shaping the genetic structure of natural populations. In addition, genetic structures of individual populations are strongly correlated with their geographical position within the species distribution area. The highest levels of genetic variation are usually found in the centre of a species' distribution and tend to decrease beyond that point. Additionally, narrowly endemic taxa are expected to be characterized by lower levels of genetic variation than their widespread congeners. To understand the historical circumstances that shape populations of sympatric and closely related taxa, microsatellite markers were used, while populations of the three closely related and sympatric Mediterranean *Salvia* species (*S. officinalis* L., *S. fruticosa* Mill. and *S. brachyodon* Vandas) served as a study model. In the populations of widespread *S. officinalis*, located in the central parts of this species' distribution area, no population genetic disturbances were detected. The narrow endemic *S. brachyodon* showed heterozygote excess, clonal reproduction and a genetic bottleneck. Because the genetic bottleneck was likely caused by the disappearance of suitable open-type habitats, the recent wildfire that cleared the terrain probably saved the *S. brachyodon* population from gradual deterioration and extinction. At the same time, clonal reproduction could serve as a valuable mechanism in the preservation of genetic variability. The results of the disjunct *S. fruticosa* population indicated heterozygote deficiency, inbreeding, hybridization with *S. officinalis* and population expansion. The hybridization with *S. officinalis* along with the abandonment of the agro-pastoral system are likely the main drivers of the strong expansion of *S. fruticosa* in the studied location. As many relevant findings and conclusions regarding historical and contemporary demography of individual populations or species can be reached only through their comparison with closely related taxa, this study demonstrates the importance and advantages of such a multi-species approach.

## Abstract (precis)

To understand the historical circumstances that shape populations of sympatric and closely related taxa, microsatellite markers were used, while populations of three *Salvia* species served as a study model. In the widespread *S. officinalis* no population genetic disturbances were detected, in the endemic *S. brachyodon* evidence for clonality and a genetic bottleneck were found while the results of the *S. fruticosa* population indicated high inbreeding levels and hybridization with *S. officinalis*. As many findings regarding demography of individual population or species can be reached only through their comparison with closely related taxa, this study demonstrates the importance of the multi-species approach.

## Introduction

There are three processes that are believed to play major roles in shaping the genetic structure of natural populations: gene flow, natural selection and genetic drift (Lesica and Allendorf 1995; Eckert *et al*. 2008). Numerous past events, either natural or human-mediated, can have substantial effects on the current intra- and interspecific genetic variability. Interspecific hybridization, genetic bottlenecks, the founder effect, inbreeding depression and rapid population expansion are just some of these effects. Genetic drift events, such as the founder effect and bottlenecks, often lead to increased rates of inbreeding, a loss of heterozygosity and the fixation of deleterious alleles (Luikart *et al*. 1998). With the increased probability of the homozygous expression of recessive deleterious alleles, the risk of extinction increases for many species undergoing population bottlenecks (Luikart *et al*. 1998). Therefore, the early detection of a bottleneck event is of central interest to conservation biologists. Among other mechanisms, hybridization plays an important role in the evolution of many taxonomic groups and can result in the formation of entirely new species. Hybridization can be of natural or anthropogenic origin; it occurs with or without introgression and can also lead to adaptive evolution and diversification via heterosis or the production of novel genotypes capable of expressing superior phenotypic traits (Stebbins 1959; Arnold 1997; Rieseberg *et al*. 2003; Seehausen 2004).

Differences and contrasting patterns in genetic structures across core, edge and disjunct populations are the source of never ending debates and research (e.g. Gaston 2003; Sagarin *et al.* 2006; Samis and Eckert 2007; Eckert *et al.* 2008). It is generally assumed that the highest abundance and the highest levels of genetic variability can be found in the geographical centre of species distribution (Surina *et al.* 2014). As we move away from the species distribution centre, population sizes gradually decrease as does genetic diversity. Similarly, a disjunct population is expected to be characterized by impoverished genetic structure as a direct consequence of its origin associated with some sort of bottleneck event followed by the absence of inter-population gene flow (Meeus *et al.* 2012). The origin of disjunct populations is usually linked to one of three possible causes: (i) human introduction, (ii) long distance dispersal and (iii) isolation after species range contractions due to either climatologically or anthropogenically induced reasons (Meeus *et al.* 2012).

The Mediterranean basin is known not only for its glacial refugium areas that have played a crucial role in postglacial recolonizations of Europe (e.g. Taberlet *et al*. 1998; Hewitt 1999, 2001; Michaux *et al*. 2003) but also for the long-lasting human presence and the influence of humans on local ecosystems that can be dated back as far as 50 000 years ago (Naveh and Dan 1973). At the beginning of the Holocene, some 10 000 years ago, this anthropogenic pressure on living systems became more intense as plants and animals were domesticated, and gradually, a sustainable agro-pastoral system developed (Miller 1992; Zeder and Hesse 2000; Blondel 2006). Consequently, the vast majority of present-day Mediterranean landscapes and accompanying species communities serve as examples of ‘coevolution’ between nature and humans that have lasted since the last glacial period. During this time, human activities and practices have shaped the complex interaction between human societies and the local ecosystems and have subsequently contributed to maintaining high levels of biological diversity (Blondel 2006; Fonderflick *et al*. 2010).

**Figure 1.**
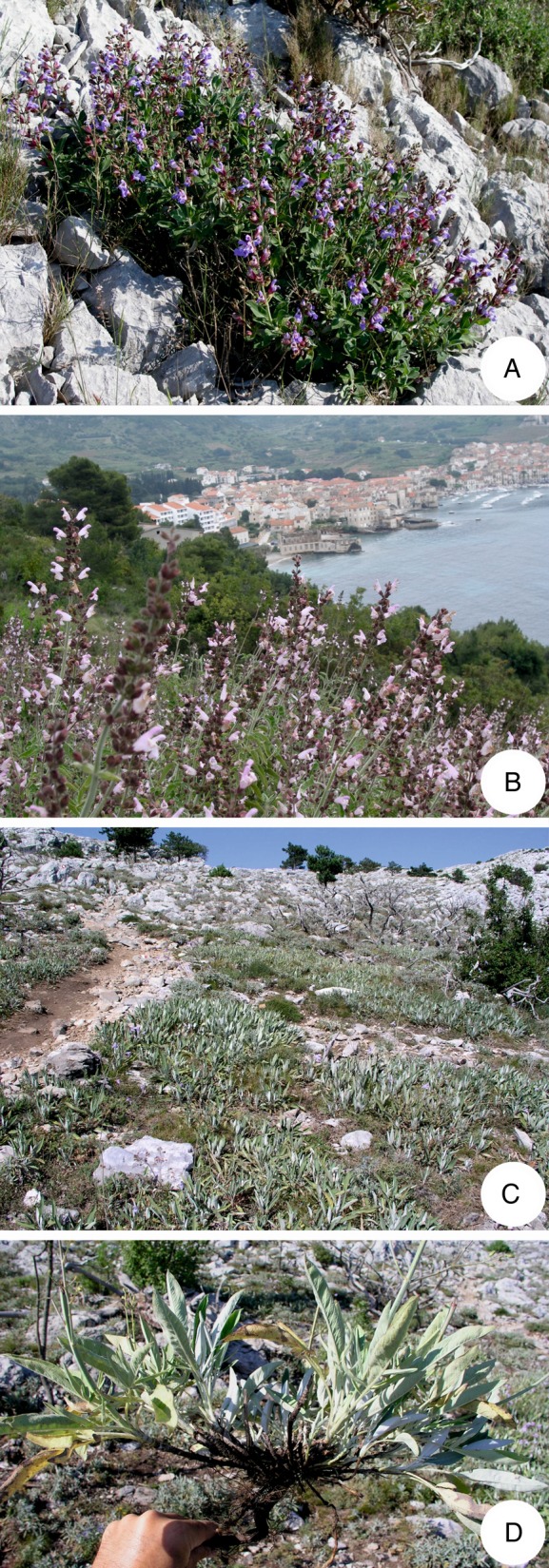
(A) *Salvia officinalis* in full bloom in the Island of Vis, (B) *S. fruticosa* in abandoned olive groves and vineyards outside the local settlement in the Island of Vis, (C) *S. brachyodon* in Pelješac Peninsula, in the burned out area. (D) Excavated *S. brachyodon* plant showing underground stolons.

With over 900 species distributed worldwide, the genus *Salvia* is by far the largest and most diverse genus in the Lamiaceae family (Walker *et al*. 2004). In Europe, 36 taxa are described and grouped into seven sections (Hedge 1972). Among them is the section *Salvia*, which comprises 13 taxa at species rank (Hedge 1972), of which *Salvia officinalis* L. (common sage) (Fig. 1) and *S. fruticosa* Mill. (Greek sage) (Fig. 1) are best known because of their economically relevant high proportions and quality of essential oil (Putievsky *et al*. 1990; Dudai *et al*. 1999). As a typical member of indigenous flora, *S. officinalis* naturally grows along the eastern Adriatic coast (Pignatti 1982), in the central and southern Apennines (Lucchese *et al*. 1995; Cutini *et al*. 2007; Di Pietro 2011), and a few relict populations can be found in continental parts of the Balkan Peninsula (Vasić 1997). The range of *S. fruticosa* extends from northeastern Libya to Sicily and southern Italy through the southern part of the Balkan Peninsula to western Syria (Hedge 1982; Karousou *et al*. 2000), but this species has been introduced to many Mediterranean countries throughout history (i.e. Malta, Spain, Portugal) by the Phoenicians and the Greeks (Karousou *et al*. 2000). In the central Adriatic region, an isolated population of Greek sage can be found on the Island of Vis (Croatia) (Flora Croatica Database; http://hirc.botanic.hr/fcd). Because this population is located ∼500 km from the nearest population of the same species in southern parts of the Republic of Albania, its origin is unclear, and it is uncertain whether this population is indigenous or naturalized, especially considering that in the 4th century BC, a well-organized Greek colony called Issa was founded at this location.

In addition to *S. officinalis* and *S. fruticosa*, in the coastal Adriatic region, a single additional member of the section *Salvia* can be found: *Salvia brachyodon* Vandas, or short-toothed sage (Fig. 1). Unlike common sage and Greek sage, *S. brachyodon* is a narrowly endemic species and can be found in only two localities: St. Ilija, the highest peak of the Pelješac Peninsula (Croatia), and Mt. Orjen, located 120 km to the southeast, in the border area of Bosnia and Hercegovina and Montenegro (Barbalić 1956; Abadžić and Šilić 1982; Šilić 1984). Because of its very limited distribution, habitat fragmentation, obvious ecological succession and other potential environmental threats (e.g. wildfires), short-toothed sage is believed to be a near-threatened species in Croatia (Flora Croatica Database; http://hirc.botanic.hr/fcd) and a highly vulnerable species in Montenegro (Petrović *et al*. 2008). Unfortunately, to the best of our knowledge, there have been no studies focused on this species for any reason other than its essential oil content (Šavikin-Fodulović *et al*. 2002; Tzakou *et al*. 2003), so numerous questions regarding the ecological status and genetic condition of this species' populations have yet to be answered.

To perform comparative population genetic research on selected species growing in an area characterized by intriguing climatological and ecological history and under strong and long-lasting anthropogenic influence (Blondel 2006; Zeder 2008), recently developed SSR markers (Molecular Ecology Resources Primer Development Consortium *et al*. 2010; Radosavljević *et al*. 2011, 2012) were applied. The goals of this study were to investigate (i) the consequences of the bottleneck effect and asexual reproduction on population genetic structure, (ii) which population genetic mechanisms could be of help in bypassing inbreeding depression, (iii) how population expansion can be detected on a genetic level and (iv) the possible consequences of inter-species hybridization. We hypothesized that: (1) selected populations of *S. officinalis* from the geographical centre of the distribution range are characterized by high levels of genetic diversity, are not suffering from any significant population genetic disturbances (e.g. accumulating mutations, genetic drift, inbreeding, population bottleneck etc.) and can consequently serve as a ‘control group’ in contrast to populations of the other two species, *S. brachyodon* and *S. fruticosa*), (2) a population of narrow endemic and endangered *S. brachyodon* is characterized by lower levels of genetic diversity than its widespread congener, i.e. *S. officinalis*, (3) a presumably non-native and disjunct population of *S. fruticosa* is genetically severely impoverished and suffers from population bottleneck, inbreeding and the absence of gene flow.

## Methods

Leaf tissue from a total of 96 individuals from four populations (24 individuals per population) was collected in two localities: Pelješac Peninsula and the Island of Vis. *S. officinalis* was collected from both sites, *S. brachyodon* from the Pelješac Peninsula and *S. fruticosa* from the Island of Vis. It is important to note that in the studied locations, *S. officinalis* occurs sympatrically with both taxa and even has an overlapping flowering period with *S. fruticosa* (late April through early May). The flowering period of *S. brachyodon* occurs in August and does not overlap with that of *S. officinalis* (I. Radosavljević, Z. Satovic and Z. Liber, pers. obs.). Voucher specimens of studied populations have been deposited in the Herbarium Croaticum (ZA) of the Department of Botany, Faculty of Science, University of Zagreb.

Genomic DNA was extracted from silica gel-dried leaf tissue by using a GenElute Plant Genomic DNA Miniprep Kit (Sigma-Aldrich, St. Louis, MO, USA). A microsatellite analysis using 13 sequence-tagged SSR loci (Table 1) was performed according to Radosavljević *et al*. (2012). All microsatellite markers were isolated from *S. officinalis* and tested for cross-amplification in *S. fruticosa* and *S. brachyodon*, but with limited success (Radosavljević *et al*. 2011, 2012). Due to this difficulty, we were unable to perform the entire analysis using a common set of SSR markers. Instead, we used three sets with the maximum possible number of common markers for each analysed combination of species. SSR amplification reactions were performed in a total volume of 20 μL containing 10× PCR buffer, 1.5 mM MgCl_2_, 0.2 mM of each dNTP, 0.075 μM TAIL FOR primer, 0.2 μM TAIL REV primer, 0.2 μM M13 primer and 0.5 U of *Taq* HS polymerase (Takara Bio Inc., Shiga, Japan). The amplification was performed with a GeneAmp PCR System 9700 (Applied Biosystems, Foster City, CA, USA) using a two-step PCR protocol with an initial touchdown cycle. The cycling conditions were as follows: 94 °C for 5 min; five cycles of 45 s at 94 °C, 30 s of annealing, beginning at 60 °C and lowered by 1 °C in each cycle, and 90 s at 72 °C; 25 cycles of 45 s at 94 °C, 30 s at 55 °C, and 90 s at 72 °C; and an 8-min extension step at 72 °C. The amplification products were run on an ABI 3730XL analyzer (Applied Biosystems) and the results were analysed with GeneMapper 4.0 software (Applied Biosystems).

**Table 1.** Population genetic parameter estimates for each microsatellite locus surveyed in four populations of *S. officinalis*, *S. brachyodon* and *S. fruticosa*. *N*_a_, number of alleles; *H*_O_, observed heterozygosity; *H*_E_, expected heterozygosity; *F*_IS_, inbreeding coefficient. Significant deviations from Hardy–Weinberg proportions after sequential Bonferroni corrections: ***significance at the 0.1 % nominal level; **significance at the 1 % nominal level; *significance at the 5 % nominal level. *N*, number of individuals; *G*, number of genotypes; Sb, *S. brachyodon* population; So1, *S. officinalis* population from the Pelješac Peninsula; So2, *S. officinalis* population from the Island of Vis and Sf—*S. fruticosa* population.

Locus name	*N*_a_	*H*_O_	*H*_E_	*F*_IS_	Sign.
Pelješac Peninsula—*S. brachyodon* (*N* = 24, *G* = 22)					
SoUZ001	4	0.750	0.691	−0.085	ns
SoUZ002	3	0.667	0.548	−0.217	ns
SoUZ005	5	0.826	0.787	−0.050	ns
SoUZ006	9	0.792	0.861	0.080	ns
SoUZ007	9	0.917	0.861	−0.065	ns
SoUZ008	3	0.833	0.659	−0.266	ns
SoUZ011	6	0.917	0.788	−0.163	ns
SoUZ014	5	0.783	0.793	0.013	ns
Mean	5.50	0.811	0.748	−0.084	*
Pelješac Peninsula—*S. officinalis* (*N* = *G* = 24)					
SoUZ001	16	0.875	0.909	0.037	ns
SoUZ003	9	0.833	0.842	0.010	ns
SoUZ007	6	0.542	0.536	−0.010	ns
SoUZ009	10	1.000	0.817	−0.224	*
SoUZ011	19	0.833	0.955	0.127	ns
SoUZ013	7	0.792	0.756	−0.047	ns
SoUZ014	15	0.958	0.913	−0.050	ns
SoUZ020	7	0.478	0.756	0.367	***
Mean	11.13	0.790	0.811	0.026	*
Island of Vis—*S. officinalis* (*N* = *G* = 24)					
SoUZ001	10	0.875	0.828	−0.057	ns
SoUZ003	7	0.583	0.652	0.106	ns
SoUZ007	6	0.783	0.717	−0.091	ns
SoUZ009	4	0.833	0.667	−0.250	ns
SoUZ011	8	0.750	0.686	−0.094	ns
SoUZ013	6	0.826	0.760	−0.087	ns
SoUZ014	8	0.870	0.840	−0.035	ns
SoUZ020	6	0.208	0.395	0.473	**
Mean	6.88	0.714	0.692	−0.033	ns
Island of Vis—*S. fruticosa* (*N* = *G* = 24)					
SoUZ003	5	0.550	0.713	0.229	ns
SoUZ005	3	0.143	0.224	0.362	ns
SoUZ007	3	0.238	0.219	−0.087	ns
SoUZ009	2	0.143	0.136	−0.053	ns
SoUZ013	3	0.500	0.567	0.118	ns
SoUZ014	2	0.191	0.176	−0.081	ns
SoUZ016	10	0.619	0.707	0.125	ns
SoUZ020	3	0.095	0.094	−0.013	ns
Mean	3.88	0.307	0.351	0.125	*
P (Sb/So1)	0.010	0.635	0.293		
P (Sf/So2)	0.012	0.005	0.016		
P (So1/So2)	0.056	0.370	0.074		

GENEPOP 4.0 (Raymond and Rousset 1995) was used to estimate population genetic parameters (the average number of alleles per locus, *N*_a_; the observed heterozygosity, *H*_O_; the expected heterozygosity or gene diversity, *H*_E_; and inbreeding coefficient, *F*_IS_) and to test the population genotypic frequencies across all loci for their conformity to Hardy–Weinberg expectations (HWE) (multilocus test). Estimates of *N*_a_, *H*_O_ and *H*_E_ in each population were compared using the Kruskal–Wallis test in SAS version 8.02 (SAS Institute Inc., Cary, NC, USA). MICRO-CHECKER v.2.2.3 (van Oosterhout *et al*. 2004) was used to check for scoring errors caused by stutters or large-allele dropouts and to estimate null-allele frequencies. BOTTLENECK program version 1.2.02 (Cornuet and Luikart 1996; Piry *et al*. 1999) was used to test for evidence of recent bottleneck events on the basis of this theoretical expectation. The expected gene diversity (*H*_E_) was compared with the expected gene diversity at mutation-drift equilibrium (*H*_EQ_) and calculated from the observed number of alleles under an intermediate two-phase model (TPM) assuming 30 % infinite allele model (IAM) and 70 % stepwise mutation model (SMM) (Di Rienzo *et al*. 1994). Based on the number of loci in our dataset, the Wilcoxon signed-rank test (Luikart *et al*. 1998) was chosen for the statistical analysis of heterozygote excess or deficiency, as recommended by Piry *et al*. (1999). The genetic distances between pairs of *S. officinalis*, *S. brachyodon* and *S. fruticosa* samples were calculated based on eight microsatellite loci by using the proportion of shared-alleles distances, *D*_psa_ (Bowcock *et al*. 1994) as implemented in MICROSAT (Minch *et al*. 1996).

To depict reticulate relationships between *S. officinalis* and *S. fruticosa* populations, a NeighborNet diagram was produced from a pairwise distance matrix with SplitsTree4 (Huson and Bryant 2006).

To determine the power of the marker set to discriminate among clones, the unbiased probability of identity (PI_unbiased_; Waits *et al.* 2001) and the PI for a population composed only by sibs (PI_sibs_; Evett and Weir 1998; Taberlet and Luikart 1999) was estimated for each locus by using GIMLET software (Valière 2002). Furthermore, to assess the probability that two samples sharing a multilocus genotype originate from distinct sexual reproductive events (i.e. from different zygotes, thus being different genets) we calculated *P*_sex_ and *P*_sex_(*f*) using the round-robin method of Parks and Werth (1993) as described by Arnaud-Haond *et al*. (2007) and implemented in GenClone ver. 2.0 (Arnaud-Haond and Belkhir 2007).

To identify putative hybrids between *S. fruticosa* and *S. officinalis*, a model-based clustering method was applied on multilocus microsatellite data using the software STRUCTURE ver. 2.3.3 (Pritchard *et al.* 2000). Ten runs per cluster (*K*) ranging from 1 to 6 were carried out on the Isabella computer cluster at the University of Zagreb, University Computing Centre (SRCE). Each run consisted of a burn-in period of 200 000 steps followed by 10^6^ MCMC (Monte Carlo Markov Chain) replicates assuming admixture model and correlated allele frequencies. No prior information was used to define the clusters. The most likely number of clusters (*K*) was chosen by comparing the average estimates of the likelihood of the data, ln[Pr(*X*|*K*)], for each value of *K* (Pritchard *et al*. 2000), as well as by calculating an *ad hoc* statistic Δ*K*, based on the rate of change in the log probability of data between successive *K* values as described by Evanno *et al.* (2005). The program STRUCTURE HARVESTER v0.6.92 was used to process the STRUCTURE results files (Earl and vanHoldt 2012).

The Bayesian method implemented by NEWHYBRIDS 1.1. (Anderson and Thompson 2002) was used to assign individuals into six classes: two pure (parental *S. fruticosa* and *S. officinalis*) and four hybrids (F_1_, F_2_ and backcrosses with the parental populations). The program was run without any prior information about the hybrid status of collected individuals, and with the uniformative Jeffreys prior option for both mixing proportions and allele frequencies. The results were based on the average of five independent runs consisting of a burn-in phase of 100 000 steps, and 500 000 MCMC sweeps. Following the suggestions of Anderson and Thompson (2002), individual genetic assignment to classes was based on a minimum posterior probability threshold (*T_q_*) of 0.50.

## Results

**Figure 2.**
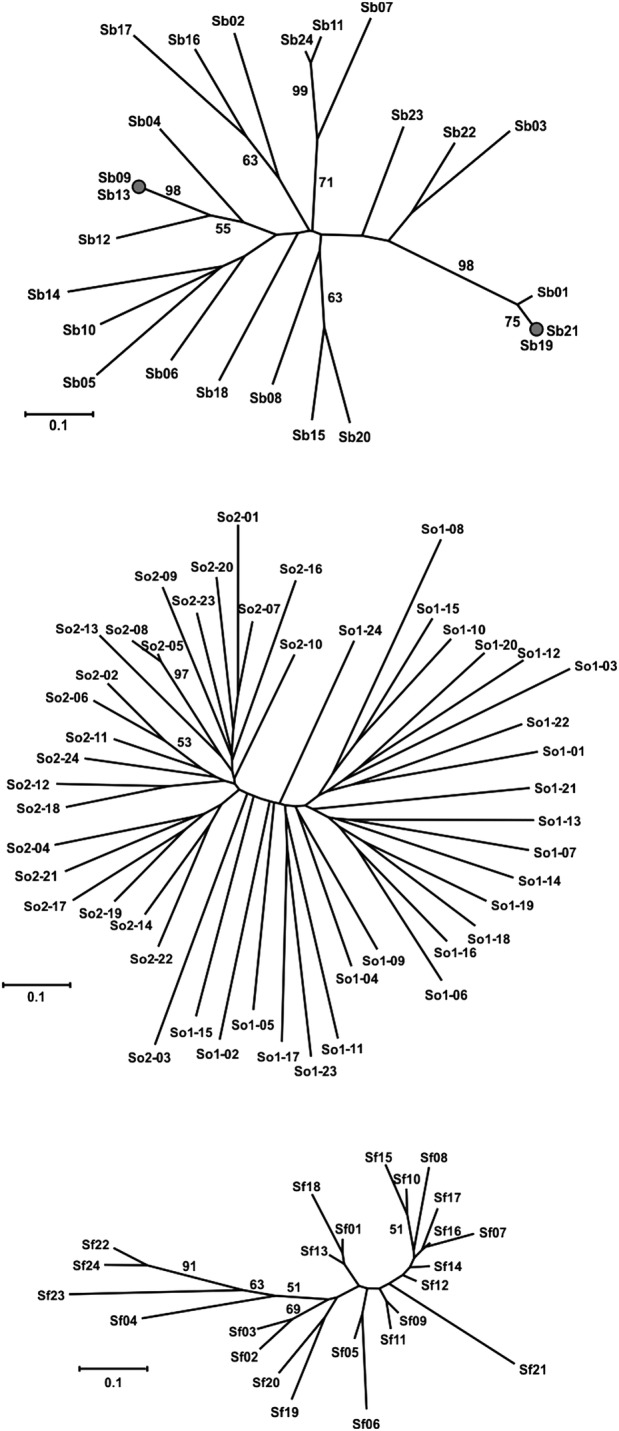
An unrooted Fitch–Margoliash tree based on the proportion-of-shared-allele distances among samples of *S. brachyodon*, *S. officinalis* and *S. fruticosa* (from top to bottom, respectively). Bootstrap support values higher than 50 % for 1000 replicates are indicated on the branches. *Salvia officinalis* samples marked as ‘So1-’ and ‘So2-’ are from Pelješac Peninsula and the Island of Vis localities, respectively.

The *S. officinalis* population from the Island of Vis showed no significant deviations from HWE, while the population from the Pelješac Peninsula was characterized by an overall *F*_IS_ value that was significantly different from zero (*F*_IS_ = 0.026, *P* = 0.029). However, a closer inspection revealed that this difference was largely explained by a highly significant *F*_IS_ value at only one locus (SoUZ020). No significant differences were noted when comparing the overall genetic parameters of these populations (Table 1). For both populations, the Wilcoxon test yielded balanced results under the TPM model, with no significant patterns of either excess or deficiency in the heterozygosity (Table 2). Low differentiation levels between *S. officinalis* populations were observed (Fig. 2).

**Table 2.** Probabilities of heterozygote excess [*P*(*E*)] and deficiency [*P*(*D*)] according to a Wilcoxon test under the TPM assuming 30 % of IAM and 70 % of SMM.

Population	Locality	TPM	TPM
		*P*(*D*)	*P*(*E*)
*S. brachyodon*	Pelješac	1.000	0.002
*S. officinalis*	Pelješac	0.680	0.371
*S. officinalis*	Vis	0.320	0.727
*S. fruticosa*	Vis	0.027	0.980

In *S. brachyodon*, the observed heterozygosity was noticeably higher than the expected (0.811 vs. 0.748, respectively), and the *F*_IS_ value was significantly different from zero (*F*_IS_ = −0.084, *P* = 0.013) (Table 1). Under the TPM, the Wilcoxon test revealed significant patterns of excess heterozygosity (*P*(*E*) = 0.002) (Table 2). Two pairs of genetically identical individuals were identified from a total of 24 collected individuals (Sb 09/13 and Sb 19/21) (Fig. 2). Eight microsatellite loci used in the study of *S. brachyodon* population were sufficiently polymorphic to allow individual identification from among 800 × 10^6^ individuals based on the estimate of the unbiased PI (PI_unbiased_ = 1.25 × 10^−9^) or among 1.286 individuals by assuming that a population was composed only of sibs (PI_sibs_ = 7.77 × 10^−4^). The probabilities of obtaining the same multilocus genotypes through distinct sexual recombination events were low in both cases (*P*_sex_ = 5.83 × 10^−7^ and 4.97 × 10^−8^, respectively), and even when using a more conservative estimate [*P*_sex_(*f*)] that takes into account possible departures from HWE, the test yielded extremely low values [*P*_sex_(*f*) = 1.04 × 10^−6^ and 1.25 × 10^−7^, respectively].

In comparison with the *S. officinalis* population from the same location, no significant differences between observed and expected heterozygosity were found.

**Figure 3.**
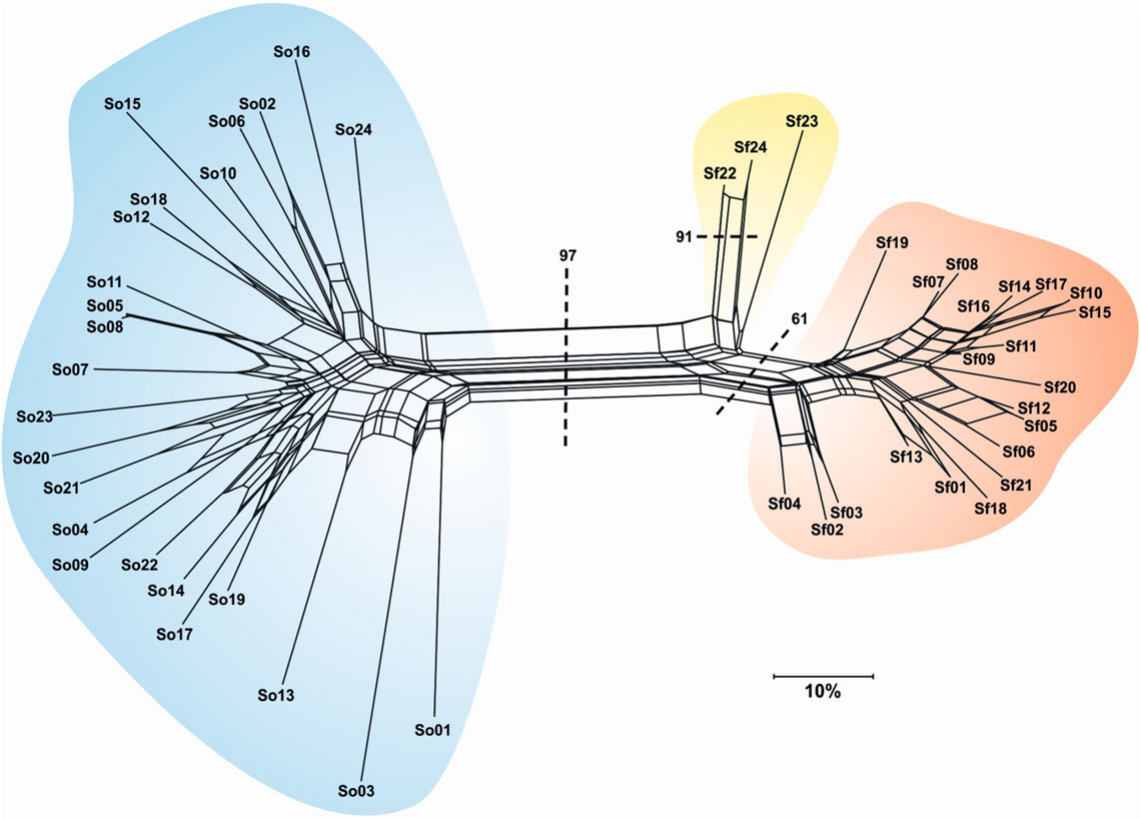
A neighborNet diagram based on the proportion-of-shared-alleles distance matrix among individuals belonging to *S. officinalis* (in blue), *S. fruticosa* (in orange) and their hybrid, *S. × auriculata* (in yellow). The bootstrap support value was derived from a Neighbor Joining analysis.

**Figure 4.**
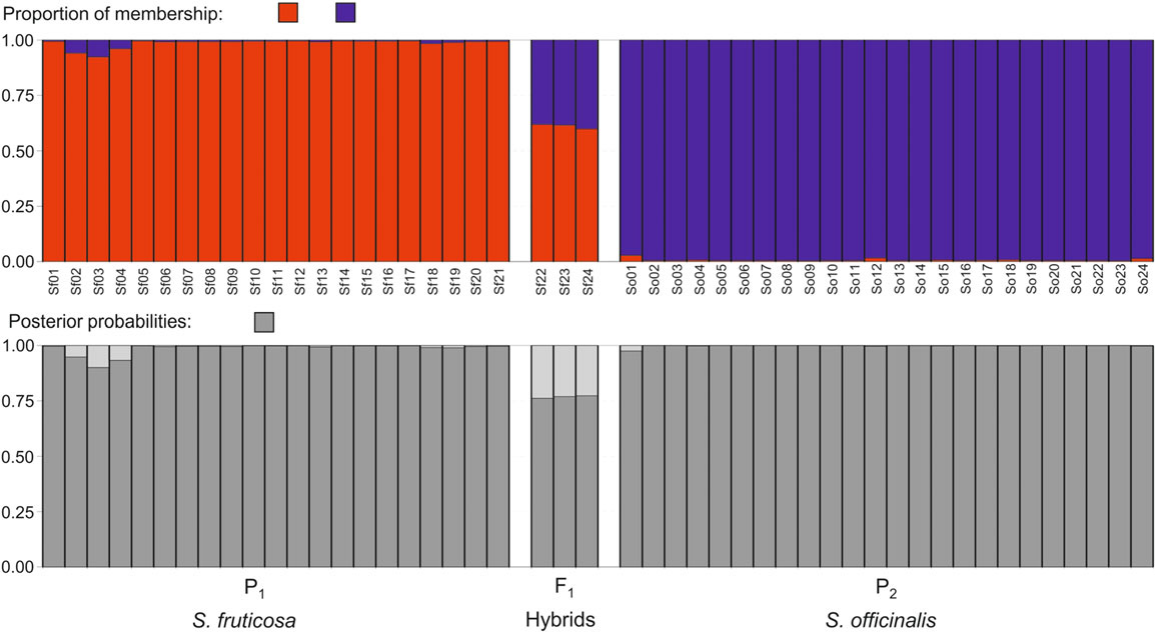
Proportions of membership of each individual in each of the two clusters as estimated by the program STRUCTURE. Each individual plant is represented by a single vertical line divided into colours. Each colour represents one cluster, and the length of the coloured segment shows the individual's estimated proportion of membership in that cluster. Assignment of individuals into classes (parental *S. officinalis* and *S. fruticosa* and F_1_) based on maximum posterior probabilities that each individual belongs to a particular class as estimated by the program NEWHYBRIDS.

In the *S. fruticosa*, the observed heterozygosity was lower than expected (0.307 vs. 0.351, respectively), which resulted in a significantly positive *F*_IS_ value (*F*_IS_ = 0.125, *P*= 0.044). Under the TPM, the Wilcoxon test revealed a pattern of heterozygosity deficiency (*P*(*D*) = 0.027) (Table 2). When comparing the overall population parameters of *S. fruticosa* and sympatric *S. officinalis*, the *S. fruticosa* presented significantly lower values of the number of alleles, observed and expected heterozygosity (*P* = 0.012, 0.005 and 0.016, respectively) (Table 1). The close genetic relationship between individuals was confirmed (Fig. 2). In addition to *S. officinalis* and *S. fruticosa*, a third group of intermediate individuals was detected, suggesting hybridization between these species (Figs 3 and 4). The results from the Bayesian analysis implemented in STRUCTURE confirmed the existence of two separate clusters (i.e. parental species) and a group of three individuals characterized by admixed proportions (Fig. 4). The highest Δ*K* was obtained at *K* = 2 (1862.93), while the second highest Δ*K* was at *K* = 4 (9.62). The presence of two well-differentiated populations belonging to parental taxa was strongly supported by the above results, as was the existence of individuals of hybrid origin.

The hybrids assignment by NEWHYBRIDS was in concordance with the results from the STRUCTURE (Fig. 4). All individuals characterized by admixed proportions were confirmed as F_1_ hybrids with very similar posterior probabilities ranging from 0.762 to 0.773. Interestingly, posterior probabilities suggesting the possible origin of these individuals through backcrossing were relatively high and ranged from 0.186 to 0.212.

## Discussion

### S. officinalis

In the *S. officinalis* from Pelješac, most loci were in HWE, with the exception of locus SoUZ011, which was characterized by a significantly positive *F*_IS_ value (Table 1). These results are consistent with the expectation that this population, located in the central part of the eastern Adriatic mainland coastal region where common sage grows abundantly, is in mutation-drift equilibrium and is not experiencing any population genetic disturbances. The results for the population from the Island of Vis were very similar; deviations from HWE were insignificant, and there were no statistically significant population genetic differences between these two populations (Table 1). Being located on an offshore island did not cause a substantial disturbance in the genetic structure of this population. Plausible explanations for this situation may lie in the fact that some 20 000 years ago, during the Last Glacial Maximum, when sea level was ∼120 m lower than today (Rohling *et al*. 1998; Clark *et al*. 2009), the Island of Vis was part of the mainland. After the sea level rose, the remaining population was simply large enough to retain most of the genetic variability that has been preserved until the present day. The low level of differentiation between the two *S. officinalis* populations (Fig. 2) is not surprising because the Pelješac Peninsula is the nearest mainland to the Island of Vis.

Being a typical heliophyte and one of the most abundant plant species in open habitats along the eastern Adriatic coast, *S. officinalis* is not threatened currently. The selected populations located in the geographical centre of the species distribution area are characterized by high levels of genetic variability and are not experiencing any population genetic disruptive circumstances. Because the results confirm hypothesis (1), *S. officinalis* populations can be treated as reference populations in contrast to populations of the other two species that probably experienced some of these disruptive factors.

### S. brachyodon

In comparison to the *S. officinalis* from the same location, *S. brachyodon* was characterized by no significant differences between the observed and expected levels of heterozygosity. There are several possible explanations for such high heterozygosity levels and heterozygosity excess (negative *F*_IS_ value). First, during the sampling expedition, a large number of bumblebees (*Bombus* sp.) were observed to visit *S. brachyodon* flowers, supporting the expectation that this species, like most sage species with large flowers (Haque and Ghoshal 1981), is predominantly an outbreeding species. Second, although the large and branched inflorescences that usually grow over 1 m in height produce a significant number of flowers, they tend to bloom sequentially (basitonic type), resulting in very few flowers on the same plant being available for pollination simultaneously (I. Radosavljević, Z. Satovic and Z. Liber, pers. obs.), thus favouring pollination between different individuals. These two traits may help to maintain genetic variation by preventing self-pollination; that is, by promoting outcrossing, the probability of creating homozygous offspring is reduced. Third, *S. brachyodon* tends not to grow individually but rather in dense groups of individuals, which indicates clonal reproduction by stolons (Fig. 1). Within our sample set of 24 individuals, two sample pairs were shown to be genetically identical (Fig. 2), thus supporting our observation. Additional conformation that clonality, rather than high inbreeding levels, is responsible for the presence of individuals sharing the same multilocus genotype, was obtained by GenClone software. With clonality being confirmed, the negative *F*_IS_ value, e.g. heterozygosity excess, could easily be explained (Delmotte *et al*. 2002; Balloux *et al*. 2003; Alberto *et al*. 2005; Ruggiero *et al*. 2005). As stated several times (Judson and Normark 1996; Welch and Meselson 2000), asexual reproduction can be responsible for maintaining high heterozygosity levels or even for increasing heterozygosity by the accumulation of mutations over generations. New mutations that occur are permanently fixed and cannot be lost through genetic drift because of the presence of non-sexual reproduction.

The BOTTLENECK test provided evidence that this *S. brachyodon* population has recently experienced severe reductions in the effective population size, i.e. a bottleneck. There are two possible explanations for a population bottleneck that should be taken into account: wildfire and ecological succession. Twelve years before sampling, in August of 1998, a devastating fire overran the entire area where this population is located (Fig. 1). However, as documented on numerous occasions, through the re-establishment of open, non-shaded habitats, periodic fires in Mediterranean-type ecosystems play a vital role in their evolutionary history (Naveh 1975; Keeley 1977; Ne'eman and Dafni 1999), rather than having a destructive impact on local biodiversity. Nevertheless, human-induced fires are prevalent in contrast to naturally occurring fires, as only 1–5 % of all the wildfires in the Mediterranean are of natural origin (Alexandrian *et al*. 1998). Thus, it is very likely that this specific fire was also human-induced. However, the presence of this isolated population suggests that *S. brachyodon*, being a native Mediterranean species, is well adapted to such an extreme event and it is unlikely that the fire caused any significant contraction in population size. Although the species cannot be characterized as a hemicryptophyte but rather as a chamaephyte, due to its clonal reproduction and the presence of underground stolons, it is also likely that resprouting is a possible mechanism of post-fire regeneration (Naveh 1975; Keeley 1977; Lloret *et al*. 2003). It is also possible that *S. brachyodon* is, such as *S. fruticosa*, an obligate seeder (Ne'eman and Dafni 1999), and may be dependent on a soil-stored seed bank. For such species, in the absence of human activities that support open-type habitats (e.g. certain styles of agriculture), occasional fires are needed to ‘clear’ the terrain of vegetation, consequently allowing seedlings to establish (Ne'eman and Dafni 1999; Santana *et al*. 2012). Once pastures, today the karstic plains in the Pelješac Peninsula are mostly abandoned; consequently, gradual ecological succession towards the indigenous black pine (*Pinus nigra* Arnold) forest (Flora Croatica Database; http://hirc.botanic.hr/fcd) is common and inevitable throughout the region. Being a typical heliophyte, *S. brachyodon* requires clear, open space to thrive and the abandonment of the traditional pasture management may result in a loss of a suitable habitats. The ecological succession at this location prior to the 1998 fire is the most likely reason for this population bottleneck and recent wildfire likely saved this population from deterioration and possibly even extinction. To re-establish and maintain the desired open habitat and the associated species community that emerged from the wildfire, grazing cattle could be reintroduced in such areas (Verdú *et al.* 2000).

Although narrow endemic species in general are considered to be characterized by low levels of genetic diversity (e.g. Gitzendanner and Soltis 2000), on some occasions it has proven to be the opposite (e.g. Dolan *et al.* 1999; Martinez-Palacios *et al.* 1999; Hannan and Orick 2000). Therefore, the high levels of genetic variation found in *S. brachyodon* that failed to support the predicted hypothesis (2) are not unprecedented. Clonal reproduction can serve as a valuable mechanism to preserve genetic diversity and in spite of the detected population bottleneck, high levels of genetic variability can indeed be found in this species.

### S. fruticosa

Two parameters that are interesting for this isolated population are the positive and highly significant *F*_IS_ value, which suggests high inbreeding levels, and the significant heterozygosity deficiency as revealed by BOTTLENECK. The results additionally confirm the hypothesis of the non-native origin of *S. fruticosa* in this location. As is often the case with anthropogenic introductions, this population was most likely founded from a very limited number of individuals who were completely isolated from the source population and from any other population of their kind. The resulting genetic drift, bottleneck and inevitable breeding between close relatives subsequently led to increased homozygosity, which had a significantly higher value when compared with the sympatric *S. officinalis* population (Table 1). Furthermore, if our hypothesis was correct and assuming that this introduction was intentional due to the settlers' (i.e. Ancient Greeks) knowledge of the aromatic and health-beneficial properties of this species (Rivera *et al*. 1994), it is reasonable to assume that this population was anthropogenically restrained from expansion and was composed of a very limited number of individuals for a long period of time. During this prolonged bottleneck period, *S. fruticosa* was grown only in gardens and vineyards surrounding local settlements. The expansion of *S. fruticosa* took place just recently, in approximately the last hundred years, when much of the local population emigrated, resulting in the abandonment of traditional agriculture. This hypothesis is indeed consistent with the current distribution of *S. fruticosa* on the Island of Vis because it is found exclusively in open, non-shaded habitats of neglected and abandoned vineyards and olive groves surrounding local settlements. The fact that *S. fruticosa* is a native East-Mediterranean species has eased its adaptation to a new environment that did not differ significantly from that in its native area of distribution, allowing *S. fruticosa* to expand quickly and form a current population of a few thousand individuals. In addition, the close genetic relationship within the population that can be observed today (Fig. 2) supports the hypothesis that this population was started relatively recently by a very limited number of individuals. During this relatively short period, there was not enough time, i.e. not enough generations, for newly emerging mutations to spread through the population and to accumulate in higher frequencies. Thus, we are now observing a significant heterozygosity deficiency (*H*_E_< *H*_EQ_). It is likely that over time and within a population of this size, the frequency of new alleles, and consequently heterozygosity, will increase.

However, considering all the results obtained in this study, it is becoming clearer that favourable ecological parameters are not the only factors responsible for such a vigorous and successful expansion of *S. fruticosa*. As already mentioned, *S. officinalis* and *S. fruticosa* occur sympatrically in this location with overlapping flowering periods. It is also important to note that a putative hybrid between these species has been recorded (*Salvia* × *auriculata* Mill.) (Putievsky *et al*. 1990), but to the best of our knowledge, only as a result of artificial crossing (Putievsky *et al*. 1990; Dudai *et al*. 1999; Reales *et al*. 2004). It is also noteworthy that three sampled specimens of *S. fruticosa* were characterized by non-typical morphological traits for this species, especially the calyx shape (non-radial, slightly zygomorphic) and the calyx trichome length and density (which were more pronounced than usual). However, because this species is characterized by exceptionally high levels of morphological variability (Hedge 1972; Karousou and Kokkini 1997), these samples were considered to originate from *S. fruticosa*. As revealed by the computer programs SplitsTree (Fig. 3), STRUCTURE and NEWHYBRIDS (Fig. 4) all three doubtful individuals (Sf22, Sf23 and Sf24) were of hybrid origin. Although the results undoubtedly support the origin of these individuals through hybridization, their asymmetric positioning in the neighbour-net diagram (Fig. 3) and somewhat inconclusive results from NEWHYBRIDS (Fig. 4) also suggest their possible origin through backcrossing with one of the parental species, i.e. *S. fruticosa*. However, the sample set is not large enough for more detailed and comprehensive study of the hybridization event, so it is impossible to address some very important questions, such as (i) are the hybrids fertile, (ii) is backcrossing to either parental species indeed present and (iii) if backcrossing is present, is it symmetrical or unilateral etc. As hybridization between species is generally considered to be rare on an individual basis, a very limited number of individuals participate in hybridization events. Consequently, hybrids are rare in the population, but even a few hybrids can provide a bridge that allows a trickle of alleles to pass between species (Mallet 2005). Thus, if we leave the possibility of the backcrossing with *S. fruticosa* open, the appearance of certain low-frequency alleles in *S. fruticosa* is not surprising. As new alleles originating from *S. officinalis* reach *S. fruticosa* through backcrossing, the alleles can be found in only a small percentage of individuals. Consequently, the frequency of these genes in the new gene pool is very low. They therefore contribute considerably to increased allelic diversity but not to heterozygosity, which is consequently detected by BOTTLENECK as heterozygosity deficiency (Table 2). However, as *S. officinalis* and *S. fruticosa* are undoubtedly closely related species it is possible to assume that at least some of these rare common alleles are not necessarily the result of inter-species introgression and horizontal gene transfer, but instead share the same origin through a common ancestor. It is reasonable to assume that the high adaptability as well as the genetic and range expansion that can be observed in the *S. fruticosa* population is supported by introgression and hybridization with a successful and well-adapted member of the indigenous flora, *S. officinalis*.

In addition, naturally occurring hybridization between these species may be of significant agricultural interest. As mentioned above, artificial crossing between these species has been already performed, and comparative research should address the yield and content of essential oil from hybrid individuals of artificial and natural origin.

The results support the hypothesis (3) that the *S. fruticosa* population is characterized by very low levels of genetic variability. As a bottleneck event can be detected in a limited time frame after it happens (Peery *et al.* 2012), we were unable to recognize it. However, high levels of inbreeding accompanied by very low levels of genetic variability suggested the occurrence of a bottleneck event in the past. The inter-population gene flow, expected to be absent in remote and isolated populations, was possibly present, but on an inter-species level. Hybridization with indigenous *S. officinalis* was confirmed, but its nature and direction are still uncertain.

## Conclusions

Our initial prediction regarding the levels of genetic diversity of three sympatrically growing *Salvia* species were only partially confirmed. In accordance with expectations, *S. officinalis* populations, as true core populations, were characterized by high levels of variability, while the disjunct and isolated population of *S. fruticosa* contained significantly less genetic variation and struggled with high levels of inbreeding. The natural hybridization between *S. officinalis* and *S. fruticosa* was confirmed for the first time. To assess the nature and direction of hybridization, more extensive research including a significantly larger number of samples and additional methods should be conducted. Although morphometrical analyses alone are considered to be of limited value in hybrid identification and characterization (Rieseberg *et al*. 1993), if combined with molecular methods, e.g. SSRs, more informative and insightful results could be obtained.

In contrast to expectation, the population of the endangered and narrow endemic *S. brachyodon*, even after surviving a population bottleneck, was still characterized by surprisingly high levels of genetic diversity. Detected clonal reproduction via underground stolons could serve as a valuable mechanism and adaptation in preservation and even accumulation of emerging mutations, although a larger sample set and appropriate analysis methods are needed for more comprehensive results and conclusions. Detailed spatial-genetic analysis would give an inside perspective on this crucial life trait (i.e. clonality) as numerous questions dealing with this species' historical and contemporary demography could be answered.

Some of the detected fluctuations in genetic structures of the studied *Salvia* populations could also be a result of certain human activities or their absence. However, before reaching any conclusions, caution is needed, as some detected genetic traits could easily be a result of specific differences in species life history traits, rather than a consequence of human activities. To more reliably address this and similar topics, a different approach is needed.

This research demonstrates the importance and advantages of a multi-species approach in population genetics studies, as some relevant findings and conclusions about each species can be reached only if a comparison among different, yet similar and closely related species is performed. The selection of the ‘control’ group should be carefully considered, as it will set the frame of the entire research. Presumably, widespread species with no significant population genetic disturbances, highly specific life traits or large-scale demographic fluctuations, should be considered as the right choice for such a purpose.

## Sources of Funding

This study was supported by the Croatian Science Foundation within the framework of the Project No. 09.01/246 (Epigenetic vs genetic diversity in natural plant populations: A case study of Croatian endemic *Salvia* species).

## Contributions by the Authors

I.R. performed the experiment, wrote and edited the manuscript and created all the figures. Z.S. conducted statistical analyses. Z.L. conceived the idea for the research and extensively edited the manuscript. All authors were equally involved in sample collection.

## Conflict of Interest Statement

None declared.
